# Can facility-based transitional care improve patient flow? Lessons from four Canadian regions

**DOI:** 10.1177/0840470421995934

**Published:** 2021-03-10

**Authors:** Sara A. Kreindler, Ashley Struthers, Noah Star, Sarah Bowen, Stephanie Hastings, Shannon Winters, Keir Johnson, Sara Mallinson, Meaghan Brierley, Mohammed Rashidul Anwar, Zaid Aboud, Jenny Basran, Leah Nicholson Goertzen

**Affiliations:** 1 Department of Community Health Sciences, 423134 University of Manitoba, Winnipeg, Manitoba, Canada.; 2 George and Fay Yee Centre for Healthcare Innovation, 8666 Winnipeg Regional Health Authority, Winnipeg, Manitoba, Canada.; 3 Applied Research and Evaluation Consultant, Centreville, Nova Scotia, Canada.; 4 Health Systems Evaluation and Evidence, 3146 Alberta Health Services, Calgary, Alberta, Canada.; 5 Department of Medicine, 12371 University of Saskatchewan, Royal University Hospital, Saskatoon, Saskatchewan, Canada.

## Abstract

Units providing transitional, subacute, or restorative care represent a common intervention to facilitate patient flow and improve outcomes for lower acuity (often older) inpatients; however, little is known about Canadian health systems’ experiences with such “transition units.” This comparative case study of diverse units in four health regions (48 interviews) identified important success factors and pitfalls. A fundamental requirement for success is to clearly define the unit’s intended population and design the model around its needs. Planners must also ensure that the unit be resourced and staffed to deliver truly restorative care. Finally, streamlined processes must be developed to help patients access and move through the unit. Units that were perceived as more effective appeared to have satisfactorily addressed these population, capacity, and process issues, whereas those perceived as less effective continued to struggle with them. Findings suggest principles to support optimal design and implementation of transition units.

## Introduction

Patient flow—the smooth, unimpeded movement of patients through settings of care—is a challenge for health systems around the world.^1^ Countries with a rapidly aging population face additional challenges as many patients may no longer require acute medical care but are not functionally ready for discharge. One response to this situation is to develop units providing transitional, restorative, intermediate, or subacute care to low-acuity hospital patients. Such interventions include transitional care units,^2^ geriatric evaluation and management units,^3^ nursing-led units providing intermediate care,^4^ and many other variants; there being no accepted collective term,^5^ we will refer to them as “transition units.” Typically, such units are designed to increase the proportion of older patients discharged home rather than to institutional settings and may also aim to expedite discharge and reduce readmissions. Thus, they have important potential benefits both to patients (by promoting return to independent living) and to system flow (by reducing pressure on nursing home and/or hospital beds). As the COVID-19 pandemic has intensified public and health system interest in developing appropriate care responses for the elderly patients, this intervention may be of particular interest to many health organizations.

Diverse types of transition units have shown evidence of improving patients’ functional status^2–4,6,7^ and reducing the risks of institutionalization^2–4^ and readmission.^4,8^ However, effects have not been observed consistently across studies.^7–11^ Results for hospital length of stay are mixed, with a few studies reporting decreases, several increases, and several no effect.^2–4,7,10,11^ The reasons why transition units may vary in effectiveness remain unclear. The literature does not facilitate comparison among models or features: transitional interventions are diverse and inconsistently defined (even interventions sharing a name may be heterogeneous), and articles do not always provide thorough descriptions of the unit studied. Thus, while a broader literature attests to the effectiveness of geriatric and restorative interventions in general,^12,13^ studies specific to transition units suggest unexplained variability in outcomes. Furthermore, there is a dearth of research on organizations’ real-world experiences adapting and implementing such models, and very little is known about current planning, operationalization, and use of transition units in Canada.

Ultimately, the literature offers limited direction on how best to design and implement transition units, or even what health systems might expect such units to achieve. This gap presents challenges for leaders wanting to explore this option. The purpose of this research was, therefore, to explore health systems’ experience of transition units in Western Canada in order to identify guidance for system planning.

As the current literature does not paint a coherent picture of transition units and how they work, we invoked a framework that helped us engage with the topic in a systematic way. The Population–Capacity–Process (PCP) model of health service design states that effective services link a defined *population* to appropriate *capacity* through a streamlined *process*.^14^ “Population” refers to people who share a need or set of needs; “capacity” to the human and physical resources required to meet those needs; “process” to the steps that link the two. The model was derived from a study suggesting that the failure of patient-flow initiatives can often be attributed to neglect of one or more of these three domains (eg, vaguely defined population, capacity unsuited to patient needs, unreliable process for accessing services). It has subsequently been used in other literature,^15–17^ but not in regard to transition units; thus, population, capacity, and process considerations specific to this type of intervention remain underexplored. We anticipated that this conceptual lens would help us identify patterns and gain greater clarity about common success factors and pitfalls.

## Methods

This research is one component of the Western Canadian Patient Flow (WeCanFlow) study, which explored patient flow strategies across ten urban health systems spanning four provinces. Taking a critical realist orientation,^18^ the study used qualitative data to furnish explanations for observed or perceived flow outcomes at the initiative and system levels. The primary qualitative data source was interviews with 300 senior, middle, and frontline managers purposively sampled for their involvement in flow; sampling, recruitment, and data collection are fully described in a companion article.^17^ The interview guide featured open-ended questions about what had and had not worked to improve flow, yielding data on over 70 interventions spread across multiple sites. Interviews were audio-recorded, and verbatim transcripts entered in NVIVO 11.

After carefully reading transcripts, at least two coders independently applied content analysis (“bucket-coding”) to identify families of initiatives, and resolved disagreements by consensus. This sub-study used the subset of data on transition units, defined as units within a hospital or other (eg, long-term care) facility that provide a limited period of care to low-acuity patients, generally with the intent of restoration of patient function to enable discharge home. (Excluded from this definition were services delivered in the home, transitional housing for patients with mental health/addiction-related needs, discharge planning activities, and units that cohorted alternate level of care patients purely for overcapacity management.)

This sub-study was a nested multiple case study^19^ of transition units. Although transition units were the fourth most frequently referenced intervention (mentioned by about one quarter of participants and addressed in all ten regions), in only four regions did participants provide sufficient data about the same initiative(s) to support in-depth analysis; these became our cases. Participants included 48 managers spanning hospital (20), community and long-term care (12), regional (11), and multi-sector (5) roles; 91% were at or above the Director level, 81% had a clinical background (nursing, medicine, or allied health), and 63% were female.

The sub-study dataset was subjected to thematic analysis^20^ to identify perceived strengths, flaws, and outcomes as reported by participants. At least two coders analyzed all data, working independently but connecting frequently to compare interpretations and establish consensus. We used the PCP model^14^ as a sensitizing concept but allowed themes to emerge inductively, a process requiring multiple iterations. Next, we developed a description of each case, then compared cases, examining the patterns associated with different perceived outcomes. To increase trustworthiness, we also investigated whether similar or different themes emerged from data concerning transition units in other regions (22 participants).

The project received approval from the University of Manitoba Health Research Ethics Board (H2015:232), University of British Columbia Providence Health Care Research Ethics Board (H15-02062), University of Calgary Conjoint Health Research Ethics Board (REB15-3026), and University of Saskatchewan Behavioural Research Ethics Board (BEH-15-377). Participants provided written informed consent.

## Results

### Description of cases

Among the four regions explored in depth, we found significant diversity in the definition, design, and reported outcomes of transition units, as well as in the length of time each had been operating and the number and type of changes each had undergone. This diversity between cases was consistent with what was found in the larger sample (where diversity was found even within the same region and among different units bearing the same names). Although all units appeared to have a restorative aim, this aim was usually described only in general terms.

The four cases included case A, a well-established menu of transition units that was positively reviewed by all informants (n = 5); Case B, a program that, while generally positively reviewed, had undergone multiple revisions to address significant problems (n = 7); Case C, a relatively recent set of initiatives that most participants deemed ineffective (for consistent reasons; n = 11); and Case D, a region-wide implementation, still underway, whose outcomes remained uncertain (this high-profile initiative was top-of-mind for many, as reflected in the large number of participants who discussed it; n = 25).

### Issues and challenges identified

The challenges identified by participants could be grouped into three categories: defining transition units’ population, ensuring that the capacity provided was fit for purpose, and establishing well-functioning processes. Some challenges were described as current while others had been resolved as the transition unit(s) evolved. Whereas case A appeared to have landed, at some point in the past, on the right population, capacity, and process, the other cases seemed less successful. Case B appeared to have appropriately defined the population (although inappropriate patients were still being referred), but showed incomplete consensus about capacity, and lingering problems around process. Case C had apparently failed to identify its population clearly, instead defining it artificially on the basis of available capacity. As rollout continued in case D, participants had not drawn definitive conclusions about effectiveness; at all events, the organization appeared to continue to struggle with definitions of population, and many challenges remained around capacity and process. (A table of illustrative quotations for each theme, by case, is available from the authors.) We note that data from non-case regions, while echoing these themes and yielding no new ones, featured different nuances (eg, one participant emphasized the wastefulness of provider-driven services that ignored patients’ goals).

#### Population

Across the sample, participants emphasized the primacy of clearly defining the population(s) to be served. At first glance, the target population for transition units may seem obvious; however, organizations’ experiences revealed otherwise. The primary explanation offered for case C’s failure was that the population had been defined arbitrarily—largely on the basis of what capacity happened to be available—resulting in artificial categories that excluded many potentially appropriate patients. In case D, the definition of “transitional care” patients seemed unproblematic but that of “subacute” patients was widely criticized as too imprecise to guide capacity planning. Only case A drew praise for population definition, having identified and addressed diverse categories of previously unmet need (even so, one participant countered that needs could be identified more proactively).

Beyond issues of population definition, participants in cases B and D noted the need to enforce such definitions, to prevent hospital staff from deliberately referring inappropriate patients to ease capacity pressures.

#### Capacity

All cases had experienced issues related to ensuring adequate and appropriate capacity; only in case A did these seem to have been resolved after early adjustments. Two main categories of issues were identified: getting the right *number* of different kinds of beds (eg, acute/subacute, general/specialized) and providing the *type* of care needed (ie, restorative). Some capacity issues (especially in case C) appeared to stem from improper population definition, but not all; even a properly defined population may be assigned improper capacity. Issues of capacity may be more complex for transition units than for other flow initiatives: not only must the total staffing complement be adequate and the staff mix appropriate (eg, rehab specializations), but providers must espouse a *restorative philosophy*. Such a philosophy promotes care that is directed toward patient-identified goals; without it, care may be provider-driven or merely custodial.

#### Process

All four cases reported experiencing issues related to process, although in case A, processes appeared to have become well-established and streamlined, requiring only occasional minor adjustments. Commonly reported issues included cumbersome processes (especially for admission); pathways of care that increased transfers of frail seniors; poor integration with other parts of the care system; and poor communication with patients, families, and care providers. As well, a few participants suggested that identified problems could have been obviated had providers been consulted during the design phase.

## Discussion

The PCP model proved to be a useful framework for analyzing strengths and limitations of organizational transition unit initiatives. Failure to appropriately define and plan for the *population*, failure to provide adequate *capacity* to support the aims of the unit, and lack of clear and collaborative *processes* were all associated with lack of perceived effectiveness and ongoing provider frustration.

Our research identified massive diversity in the conceptualization and operationalization of transition units, and the four cases studied in depth were at different stages of development. Nonetheless, some clear take-away messages emerged. First, it is essential for organizations to begin by analyzing population needs (which may require detailed data collection on unmet need) and defining, on this basis, the unit population and eligibility criteria. Without a clearly defined population, there will not be appropriate capacity; without solid population–capacity matching, there will not be good processes. Across regions, it was consistently found that getting design and implementation right take considerable time and effort; however, failure to adequately address population definition upfront appeared to result in an unnecessarily difficult and protracted period of growing pains.

Second, findings highlighted the importance of instituting, and maintaining fidelity to, a restorative philosophy. Transition units are not merely a flow intervention, but also, explicitly, an intervention to improve patient functioning and independence. However, pressure to focus on overcapacity management and cost containment (reported as part of the context in all regions studied) may result in inattention to crucial design elements—or (even in well-designed initiatives) failure to maintain a focused response on restoration. Our research also suggests that evaluation of transition units requires thoughtful consideration of priority outcomes, ensuring they reflect program aims and a restorative philosophy of care; for example, should we emphasize improvement in patient function or length of stay? What about establishment and attainment of patient goals? What institutionalization rate is indicative of success? Another question to consider is whether a restorative approach should begin in the acute phase of care, as in the Acute Care for Elders model^12^; at the least, acute units should shoulder their responsibility to prevent deconditioning,^13^ rather than view patient function as the sole purview of transition units.

Finally, it is important to ensure that the process of accessing a transition unit does not expose patients to additional delays and hazards. Evaluations should encompass transitions into and out of the unit, and direct entry (from the community or emergency department) is a potentially promising option.^21^

This research has several limitations. First, it does not encompass all experiences with transition units across (Western) Canada. Our sample of four contrasting cases, while not unusual for the study design, is smaller than it might have been had data collection focused solely on transition units. During the broad, open-ended interviews, participants tended to focus on particularly recent, successful, or problematic initiatives; some interventions with a long-standing role in a jurisdiction’s flow strategy (eg, subacute care in Alberta) were barely mentioned. Intervention-specific interviews would have yielded more data beyond (perhaps even within) the four selected cases and might have suggested additional themes; further research might also expand to a national scope.

Second, as quantitative data on unit outcomes were unavailable, we were limited to participants’ subjective assessments, which must be interpreted cautiously. Indeed, we noted some inconsistencies in such assessments (principally in case B, although this might be attributable to participants having been involved in the unit at different times). Third, the sample was restricted to management; frontline providers, patients, and caregivers would have enriched the data with potentially contrasting perspectives.

Although this preliminary research was not able to evaluate particular models or specific implementation processes, analysis of these four cases does identify key issues affecting perceived effectiveness of interventions, as well as suggest emerging principles (based on both successful and less successful actions) that might guide planning and implementation of transition units in diverse settings.

## Conclusion

Development of a range of effective strategies to facilitate patient flow is sure to remain a priority for healthcare managers in the years to come. Creation of transitional units for specific low-acuity patients is one category of response that also promises benefits in terms of avoiding institutionalization or readmission. If transition units are to be effective, they must first ensure that the population the unit is intended to serve is clearly defined and that the intervention is designed around their needs. These interventions must then be ensured of adequate and appropriate capacity to achieve their restorative goals, and effective processes must be implemented to allow patients smooth passage into and through the unit. Attention to these design elements may help healthcare organizations realize the potential of transition units.
